# Immune response following traumatic spinal cord injury: Pathophysiology and therapies

**DOI:** 10.3389/fimmu.2022.1084101

**Published:** 2023-01-06

**Authors:** Robert C. Sterner, Rosalie M. Sterner

**Affiliations:** ^1^ School of Medicine and Public Health, University of Wisconsin-Madison, Madison, WI, United States; ^2^ Department of Laboratory Medicine and Pathology, Mayo Clinic, Rochester, MN, United States

**Keywords:** traumatic spinal cord injury, SCI, stem cell, extracellular vesicle, vesicle, neuroimmunology, tSCI therapies

## Abstract

Traumatic spinal cord injury (SCI) is a devastating condition that is often associated with significant loss of function and/or permanent disability. The pathophysiology of SCI is complex and occurs in two phases. First, the mechanical damage from the trauma causes immediate acute cell dysfunction and cell death. Then, secondary mechanisms of injury further propagate the cell dysfunction and cell death over the course of days, weeks, or even months. Among the secondary injury mechanisms, inflammation has been shown to be a key determinant of the secondary injury severity and significantly worsens cell death and functional outcomes. Thus, in addition to surgical management of SCI, selectively targeting the immune response following SCI could substantially decrease the progression of secondary injury and improve patient outcomes. In order to develop such therapies, a detailed molecular understanding of the timing of the immune response following SCI is necessary. Recently, several studies have mapped the cytokine/chemokine and cell proliferation patterns following SCI. In this review, we examine the immune response underlying the pathophysiology of SCI and assess both current and future therapies including pharmaceutical therapies, stem cell therapy, and the exciting potential of extracellular vesicle therapy.

## 1 Introduction

Traumatic spinal cord injury (SCI) is severely debilitating and is associated with substantial financial and emotional costs (1–3). In the United States, >12,000 patients annually suffer from SCI, with approximately 270,000 SCI patients across North America (1, 3, 4). The pathophysiology of SCI is complex with the initial trauma (primary injury) causing acute cell dysfunction/death that is further propagated by secondary injury cascades (5, 6). Among the secondary injury mechanisms, overactivation of the systemic immune response and neuroinflammation are key determinants of the extent of injury (6, 7). More specifically, the inflammatory cascade following SCI is complex and involves both the adaptive and innate immune responses, several cell types, and many inflammatory cytokines including interleukin-1β (IL-1β), interleukin-6 (IL-6), and tumor necrosis factor alpha (TNFα) (8, 9). Although the immune response following SCI also has many beneficial effects, it is thought that the large-scale inflammatory response is a key factor in causing neural degeneration (8, 9). Management of traumatic spinal cord injury (tSCI) patients has mainly focused on the timing of surgical decompression in which recent studies have demonstrated that early surgical decompression of SCI patients within 8-12 hours following SCI is associated with improved neurological outcomes based on American Spinal Injury Impairment Scale (AIS) improvements (10, 11). Given the pivotal role the immune response plays in dictating the extent of secondary injury and neural degeneration, selectively targeting the immune response to SCI to promote neural regeneration instead of degeneration is a premier therapeutic target to improve functional outcomes (12). In order to develop neuro-immunological therapies for SCI, however, a detailed characterization of the immune response following SCI is necessary. Over the last two decades several studies have characterized the complex cytokine/chemokine cascades and cell infiltration patterns (7). In this review, we examine the immune response including the cytokine/chemokine cascades as well as the cell proliferation patterns underlying SCI and evaluate current and future therapies including pharmaceutical therapies, stem cell therapy, and extracellular vesicle therapy.

## 2 Pathophysiology of SCI

### 2.1 Primary versus secondary phases of injury

**Figure 1:**
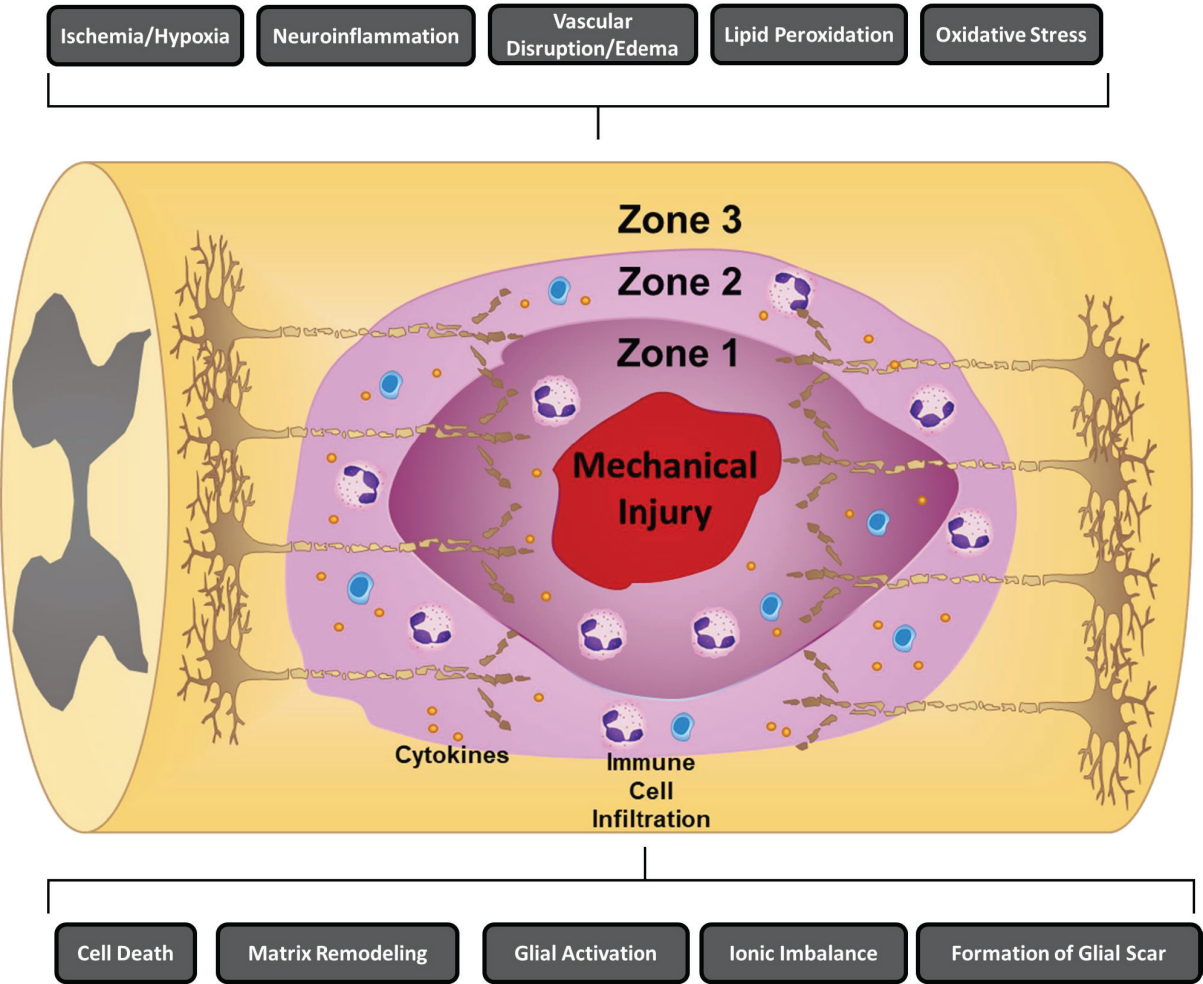
Mechanisms of secondary injury following traumatic spinal cord injury. Several key secondary mechanisms of injury that have been shown to contribute to the propagation of acute cell dysfunction and cell death including ischemia/hypoxia, neuroinflammation, vascular disruption/edema, lipid peroxidation, oxidative stress, cell death, ionic imbalance, formation of a glial scar, glial activation, and matrix remodeling are depicted. Following SCI, three zones that differ in tissue quality form including: (1) Zone 1, which is mainly a product of the initial trauma and is defined by regions of necrosis, inflammation, and cysts; (2) Zone 2, is characterized by regions of incomplete injury that are still accompanied by immune cell infiltration, axonal swelling, and Wallerian degeneration; and (3) Zone 3, which are histologically intact areas (15, 16).

The pathophysiology underlying SCI occurs in two phases, the primary and secondary phases of injury. The primary injury phase is caused by the initial mechanical forces delivered to the spinal cord at the time of injury (5, 6). These mechanical forces cause direct structural damage to the surrounding neuronal tissue and vasculature tissue resulting in acute cell dysfunction and cell death (5, 6, 13). Broadly, four types of primary injury mechanisms have been described: (a) impact plus persistent compression; (b) impact alone; (c) distraction; and (d) laceration/transection (5, 6). Among the four mechanisms of primary injury, impact plus persistent compression is most common and frequently occurs *via* burst fractures (5, 6). In contrast to the primary phase of injury which occurs within a short window of time, studies have shown that the secondary phase of injury continues for days to weeks or even months after SCI (12–14). The secondary phase of injury propagates the acute cell dysfunction and cell death initially caused by the primary injury. Several secondary injury mechanisms (**Figure 1**) have been shown to contribute to propagation of acute cell dysfunction and cell death including neuroinflammation, ischemia, free radical formation, lipid peroxidation, blood CNS barrier break down, edema, release of proteases, and excitotoxicity (12–14). Importantly, the immune response plays a profound role in the propagation of secondary injury after SCI.

Following SCI, three zones that differ in tissue quality form including: (1) Zone 1, which is mainly a product of the initial trauma and is defined by regions of necrosis, inflammation, and cysts; (2) Zone 2, is characterized by regions of incomplete injury that are still accompanied by immune cell infiltration, axonal swelling, and Wallerian degeneration; and (3) Zone 3, which are histologically intact areas (15, 16). Among the zones, Zone 1 is the region that has the highest level of damage with the lowest chance of neuron survival/recovery (12, 15, 16). Neuroinflammation has been shown to be a critical secondary injury mechanism that directly alters the progression of lesions and cell function across all three zones (12). Although surgical decompression is the first line therapy for spinal cord compression following SCI, studies have shown that the severity of injury is dependent on the extent of secondary damage (10, 12, 17). Therefore, therapeutic strategies have focused on strategies to modulate the immune response after SCI in order to promote neuro-regeneration and neuroprotection. This review will focus on examining the immune response underlying the pathophysiology of SCI and assesses both current and future neuro-immunological therapies.

### 2.2 SCI induced immune depression versus autoimmunity

After SCI, both immune depression and autoimmunity commonly co-occur (18, 19). The CNS exerts control over the immune system through several pathways including the hardwired fibers of the autonomic nervous system (19, 20). Throughout the body, both central and peripheral autonomic nervous system sensors function to relay information about the status of the body’s immune system (19, 20). Due to SCI, the CNS interaction with the immune system is disrupted resulting in a significant systemic decrease in immune function with reports of several different functional alterations in macrophages, T and B cells, and natural killer (NK) cells (20, 21). This systemic decrease in immune function after SCI is termed spinal cord injury-induced immune deficiency syndrome (SCI-IDS) (12, 21). Although the precise mechanism(s) causing SCI-IDS are not completely clear, patients with SCI-IDS are at a significantly higher risk of developing complications such as infections, pneumonia, and urinary tract infections (12, 21).

One recent study suggested that the mechanism underlying post-SCI immune suppression may be due to the fact that after SCI, significant plasticity develops below the injury site within the autonomic spinal circuitry resulting in a sympathetic anti-inflammatory reflex (22). Furthermore, the authors showed that chemogenetic silencing of this reflex prevents post-SCI immune suppression and could be a promising therapeutic approach in the future (22). Although there are several negative effects of SCI-IDS, immunosuppression following SCI may actually be advantageous and serve a protective function (12, 23). SCI damages the blood-spinal cord barrier and spine parenchyma, which exposes cells of the adaptive immune system including B and T lymphocytes to CNS antigens (12, 23). Exposure of the adaptive immune system to CNS antigens can then trigger autoimmune responses and the production of autoantibodies that have been shown to worsen pathology within the spinal cord (12, 23). Notably, studies in mice suggest that SCI especially impairs the production of new antibody responses but preserves existing immunity (18, 24). Thus, autoantibodies identified after SCI may have existed prior to the injury (18). This hypothesis was supported by a study that demonstrated a set of naturally occurring auto-antibodies expanded following SCI (18). Although it is clear that trauma induced autoimmunity has the potential to contribute pathologically, the triggering mechanisms and molecular signatures will require further investigation in the future.

### 2.3 Inflammatory cascade after SCI

**Figure 2:**
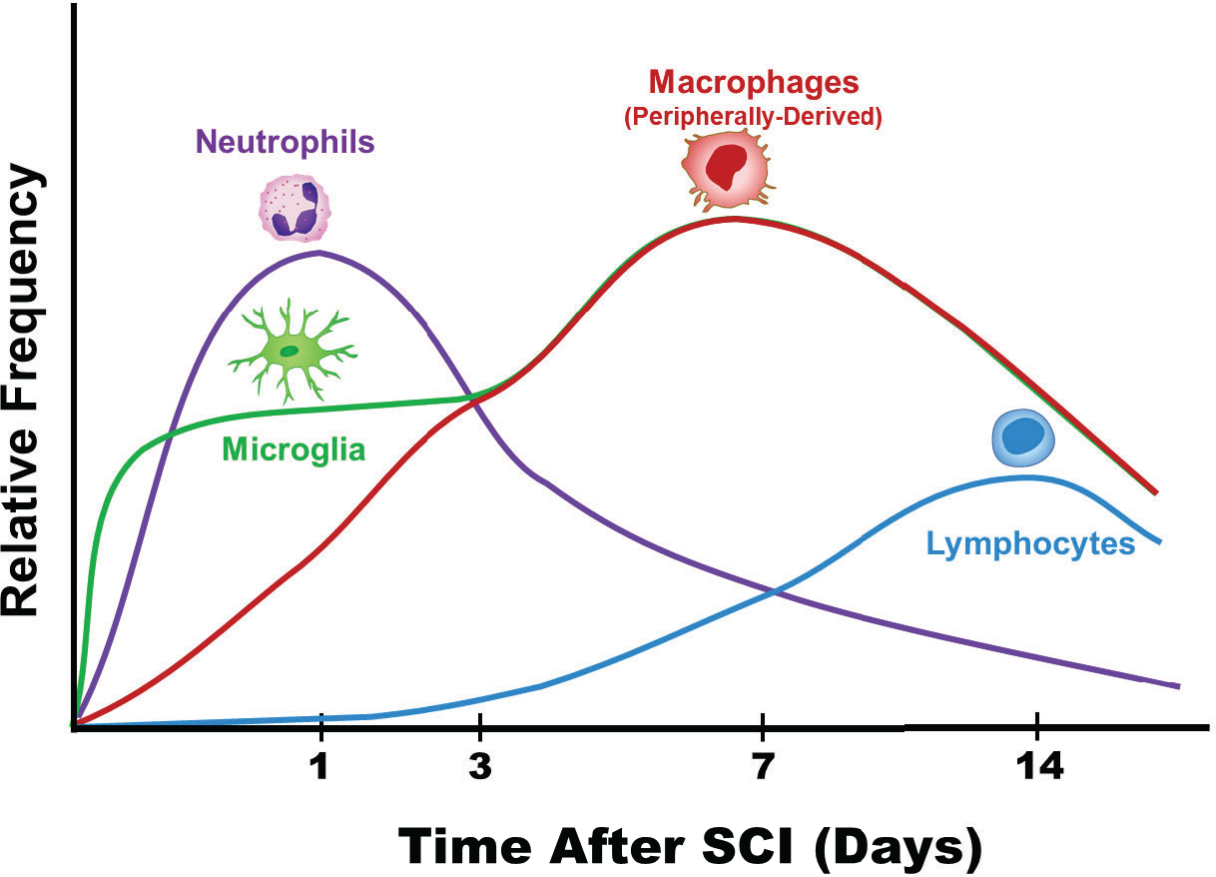
Patterns of immune cell infiltration following spinal cord injury. The order and timing (7, 27–31) of various immune cell infiltrates is depicted (32). The level of neutrophils peak at approximately 24 hours after SCI and decrease over the course of the next 7-10 days. Comparatively, lymphocytes peak much later and at lower levels compared to neutrophils. The resident macrophages, microglia, are early responders after SCI. Eventually, microglia become indistinguishable from the peripherally derived macrophages from a morphological standpoint.

Following CNS injury, neuroinflammation is a result of innate immune system activation that is mediated by cytokines and chemokines released by astrocytes, resident microglia, endothelial cells, and peripherally derived immune cells (14). Several studies have clearly demonstrated that within hours of CNS injury, the inflammatory cytokines IL-1, IL-6, and TNF are all upregulated (7, 25). Upregulation of these potent inflammatory cytokines subsequently triggers substantial infiltration of macrophages, microglia, and neutrophils (7). Furthermore, these infiltrating immune cells continue to produce and secrete additional chemokines and cytokines that modulate the immune response. The size of the primary insult is a critical factor in determining the magnitude of neuroinflammation (26). In the case of SCI, additional cell death is often caused by overactivation of the immune response. Therefore, in order to develop therapies that can selectively dampen the overactive immune response, the immune response following SCI must be thoroughly characterized. In this review, we briefly review the literature on the systemically upregulated cytokines derived from the blood/cerebrospinal fluid (CSF), which may have clinical utility as biomarkers and focus on the timeline of the local cytokine/chemokine cascade and cell infiltration patterns (**Figure 2**) (32).

### 2.4 Cytokine patterns from serum and CSF as biomarkers

Overall, the patterns of cytokine and chemokine upregulation following SCI are generally similar in the serum and CSF compared to the patterns within the injured tissue itself. Understanding the tempo and the major players of the immune response in SCI could allow for the identification of biomarkers from patient’s serum and CSF that could guide management of SCI. Within 6 hours after SCI, a rat model showed that CSF levels of IL-1, IL-10, IL-17*a*, IFN-γ, and TNFα were all significantly elevated (7, 33). Furthermore, the serum levels of IL-1β, IL-6, and TNFα, remained elevated throughout the first week after SCI in rats (7, 34, 35). A clinical study examining changes in serum biomarkers found that within 24 hours after SCI, there were significant elevations in structural proteins including tau, S100β, and GFAP as well as elevations in the cytokines IL-6, IL-8, and MCP-1 (7, 36). One study reported decreased serum levels of IL-1β and IL-10 after 14 days following SCI while another study found persistently elevated IL-1β and no change in IL-10 28 days after SCI (7, 36). Notably, this study (36) also reported persistent serum elevations in TNFα 28 days after SCI and downregulation of IL-4 at 28 days. Importantly, clinical studies have revealed that lower levels of serum and CSF IL-6, IL-8, tau, S100b, GFAP, and MCP-1, are associated with significantly higher AIS grade improvement and neurological recovery compared to patients that did not improve (37, 38). In these studies, the most severely injured patients typically had the highest levels of NSE tau, S100b, and GFAP (37, 38). In the future, additional clinical studies will be needed to further validate these proteins as true biomarkers that could enhance clinical decision-making.

Below, we discuss the timeline of the inflammatory cascade following SCI including cytokine/chemokine regulation and cell infiltration patterns (**Figure 2**).

## 3 Timeline of the cytokine/chemokine cascade and cell infiltration

Within the first 24 hours following tSCI, proinflammatory cytokines including TNFα, IL-1β, and IL-6 appear to be upregulated and are likely the key mediators of injury (25, 33, 39–45). Studies have also suggested that other cytokines including IL-7, growth-related oncogene (GRO), macrophage inflammatory protein 1-alpha (MIP-1α), and monocyte chemoattractant protein 1 (MCP-1) are upregulated while IL-4, IL-10, IL-13, and TGF- β1 remain at baseline levels within the first 24 hours after tSCI (33, 43, 46). Here, we provide an in-depth review of the patterns of cytokine/chemokine regulation and cell infiltration patterns within the first 24 hours and examine the inflammatory cascade over the course of the first two weeks following tSCI.

### 3.1 1 Hour after SCI

Within minutes after the primary SCI, secondary injury mechanisms begin to cause significant damage (7, 47, 48). More specifically, edema, oxidative damage, cell permeabilization, excitotoxicity (glutamate), ischemic injury due to microvascular supply destruction, and proapoptotic signaling among other factors all contribute to substantial cell dysfunction and cell death (7, 47, 48). Dysregulation in intracellular calcium also causes injury and further cell death as dysregulated intracellular calcium results in calpain activation causing mitochondrial dysfunction (7, 48–50).

Although in the early phases of injury the resident immune cells of the CNS, microglia, respond in a protective manner, quickly microglia morph into proinflammatory cells that secrete cytokines that trigger peripheral immune cell infiltration (27, 51–53). For example, one study showed that microglia and astrocytes start expressing TNFα mRNA and IL-1β within a half hour following SCI (25). Notably, the number of TNFα positive cells peaked 1 hour following SCI (25).

### 3.2 1-3 Hours after SCI

Within 1-3 hours following tSCI, TNFα, IL-1β, and IL-6 are believed to be the key mediators. mRNA colocalization studies have demonstrated that microglia, neurons, and astrocytes synthesize TNFα, IL-1β, and IL-6 during the early hours after SCI in a mouse model (7, 25). In five other studies, the levels of TNFα in the early hours after SCI were also substantially increased (39–41, 54, 55). Three of these studies showed a significantly elevated level of IL-1β during the early hours of SCI; however, two studies using enzyme-linked immunosorbent assays (ELISAs) reported that there was not an increase in IL-1β during the early hours of injury (34, 39–41, 54). Hellebrand et al. (7) suggested that this inconsistency may be due to the time necessary to synthesize the full protein product as well as the time required for caspase 1 to proteolytically process it to its active form. Studies assessing IL-6 upregulation observed similar results to the IL-1β pattern (39–41, 54).

### 3.3 3-6 hours after SCI

Although there appear to be a few differences in the adaptive immune system among rodents, a majority of literature has shown a significant increase in proinflammatory cytokines (7, 25, 40, 56–59). More specifically, within the window of 3-6 hours after SCI, several studies have shown that TNFα, IL-1β, and IL-6 are the primary cytokines significantly elevated (7, 25, 40, 56–59). In addition, most studies agree that within the 3-6 hours window there is a slight lag in upregulation of IL-1β and IL-6 relative to TNFα and IL-1*a* (7, 25, 40, 41, 57–59). The increase in cytokine production in microglia and astrocytes also results in increased peripheral immune cell invasion. For instance, neutrophils start to arrive within 4-6 hours after SCI and function to prepare the region for repair through the production of proteolytic and oxidative enzymes (28, 29). The overwhelming number of neutrophils, however, can cause significant tissue damage (28, 29).

At 3 hours following SCI, the number of cells expressing TNFα mRNA within the center of the injury is significantly increased relative to control cells; however, the number of cells expressing TNFα mRNA was 66% lower compared to the 1 hour time point (25). Additional evidence suggests that TNFα levels return closer to baseline following rapid onset as two studies reported no significant increase at 4 hours following SCI (40, 54).

Even though anti-inflammatory cytokines could decrease the levels of proinflammatory cytokines, studies suggest that following SCI, the levels of anti-inflammatory cytokines are either low or absent (7). Theoretically, IL-10 is an anti-inflammatory cytokine that could reduce pro-inflammatory cytokine levels and IL-4/IL-13 are anti-inflammatory cytokines that can trigger alternative macrophage activation (7, 60, 61). Studies using rodent models have shown that IL-13 was increased at 4 hours following SCI in rats but not in mice, and IL-10 was increased in mice but not in rats (40, 57, 58, 60–64). Finally, Xiong et al (65) reported increases in 4-hydroxynonenal (4-HNE), which is formed during lipid peroxidation, starting at 3 hours following SCI (7, 65).

### 3.4 6-12 after SCI

Within the 6-12 hour time period, the proinflammatory environment continues facilitating increased microglia proliferation/recruitment and increased peripheral immune cell invasion (7). Studies agree that from 6-12 hours after SCI, TNFα, IL-1β, and IL-6 continue to be upregulated while IL-4, IL-10, and IL-13 remain at baseline levels (25, 33, 39–45). In addition, other immune mediators that are significantly increased during the 6-12 hour window include macrophage inflammatory protein 1-alpha (MIP-1*a*), monocyte chemoattractant protein 1 (MCP-1), C–X–C motif chemokine ligand 1 (CXCL1), growth-related oncogene (GRO), and RANTES (Regulated upon Activation, Normal T Cell Expressed and Presumably Secreted) (33, 43, 46).

Initially, neutrophils are the most common infiltrating cell type (30). Overactivation of neutrophils can be quite destructive to tissue as these cells release substantial amounts of neurotoxic substances including chemokines, enzymes, reactive oxygen species (ROS), and reactive nitrogen species (RNS) (9, 29, 66, 67). At approximately 8 hours after SCI, apoptosis from the inflammatory response peaks in neuronal cells (68, 69).

### 3.5 12-24 hours after SCI

During the 12-24 hour window a majority of studies show: (a) upregulated levels of the proinflammatory cytokines TNFα, IL-1β, and IL-6; (b) elevated levels of IL-7, GRO, MIP-1α, and MCP-1; and (c) baseline levels of TGF-β1, IL-4, IL-10, and IL-13 (39, 45, 46, 56, 70–72). At 24 hours following SCI, the level of neutrophils peak (**Figure 2**) (31). Neutrophils infiltrating the site of injury function to clear debris, proteases release reactive oxygen species, and neutrophils secrete myeloperoxidase, elastase, and proteases (31). In glial cells, apoptosis peaks at 24 hours following SCI (68, 69).

One study in rats showed that lymphocytes start to collect near blood vessels within gray matter as early as 6 hours after SCI (52). Lymphocytes that infiltrate the site of injury produce IL-1β, IL-6, TNFα, and LIF at 12 hours post injury (25). The levels of the neurotoxic 4-HNE peak at 24 hours and for two weeks remained elevated (65).

### 3.6 24 hours-7 days after SCI

In a rat model of SCI, flow cytometry showed that the initial phase of cellular inflammation at 1 day after SCI consisted of an early neutrophil peak; at 7 days after SCI a peak of macrophages/microglia; and at 9 days after SCI a peak of T-cells (73). In mice, although neutrophils enter the site of injury at 6 hours after SCI, levels do not peak until 14 days after injury and remain for up to two weeks (27). At day 5 after SCI, phagocytic macrophages are most commonly localized in areas of necrosis while microglia are mainly at the margins (7, 74). Microglia are highly dynamic and during the first week after SCI have been shown to proliferate extensively (7, 74). Furthermore, at the border of the site of injury between infiltrating peripherally-derived macrophages and astrocytes, microglia form a dense cellular interface (7, 74). Due to the initial injury, axons start to retract at day 2 and macrophages also trigger a late phase of axon retraction (75–77).

After 2 days following SCI, co-localization studies showed that TNFα mRNA levels had returned to a level that was not significantly different from the level of mice that had received control laminectomies (7, 25). The average number of cells expressing IL-6 mRNA decreased from 24 hours-4 days after SCI to an almost zero cells 7 days after injury (7, 25). Similarly, the number of IL-1β positive cells decreased at 2, 4, and 7 days following SCI (7, 25). Notably, although the number of cells expressing the above transcripts decreased, several studies in both rats and mice have reported increased levels of TNFα, IL-1β, and IL-6 at 1, 3, and 7 days following SCI (7). In addition, GRO has been reported to be significantly increased at 1, 3, and 7 days following SCI (46, 71, 78).

During the first week of injury, studies have shown that there is a significant increase in production of chemokines that recruit T-cells, monocytes, and dendritic cells to the lesion (7). Previous studies have shown upregulation of MIP-1α at 1 and 3 days following SCI, CXCL1 expression is increased at 3 and 7 days following SCI, and RANTES is significantly upregulated at 3 days following SCI (43, 71, 78–81). While there have been some conflicting results, a majority of studies show that MCP-1 mRNA is increased in mice 1 day after SCI with return to baseline 7 days following SCI (82, 83). Among rats and mice, it is important to note that there have been some contrasting behaviors (7). Cytokines that have been reported to have at least somewhat contrasting behaviors among rats versus mice include IL-2, IL-4, IL-5, IL-13, and IFN-γ (7, 70, 80, 81, 84). A majority of studies have suggested that IL-10 is not significantly increased until 3 days following SCI although some reports have not observed changes in IL-10 levels after SCI in both mice and rats (40, 42, 79). Finally, TGF-β1 was reported to be upregulated at 3 and 7 days following SCI (7, 39).

### 3.7 7-14 days after SCI and beyond

Macrophages and peripherally derived macrophages peak at 7 days following SCI and persist for months after the injury in mice, rats, and humans (15, 60, 73, 74, 85). At 14 days following SCI injury, Mukhumedshina et al. (78) found that IL-1*a*, IL-2, MIP-1*a*, and GRO were significantly increased in rats while IL-2, IL-5, IL-13, IL-17*a*, IL-18, and GM-CSF were significantly decreased (78). In mice on the other hand, IL-1*a*, IL-4, IL-7, IL-12, IL-15, MIP-1*a*, MCP-1, and RANTES were significantly upregulated (79, 84, 86). Mice also were found to have a second surge of cells expressing IL-1β and TNFα at 14 days after SCI (25). Rats, however, did not undergo a second surge at 14 days after SCI (78, 87). Future studies examining the cytokines profiles after 14 days in SCI are necessary as there is less data compared to other time frames.

### 3.8 Cellular reactions and cytokine signaling at 14 days after SCI and beyond

Astrocytes are a subtype of CNS glial cells that function to maintain neurons and the blood spinal cord barrier (7, 88). Initially, reactive astrocytes flock to the injury site to aid in tissue repair but eventually become scar forming astrocytes (7, 88). These scar forming astrocytes form a glial scar surrounding the injury site. The layer of astrocytes around the lesion are defined by increased expression of glial fibrillary acidic protein (GFAP)/intermediate filaments, cellular hypertrophy, and process extension (7, 88). Overall, the glial scar has immune cells in the center with macrophages interacting with pericytes, which are the main scar connective tissue source around the edges and astrocytes that surround the periphery (74, 89).

The question of whether T cells mediate wound healing or result in secondary degeneration is controversial (7, 15, 90–92). T cells mediate adaptive immunity and appear to infiltrate into the spinal cord at somewhat different times depending on the species of animal (7). Studies have shown that chemokines CXCL10 and RANTES are critical for the proliferation and cytokine production of T cells (7, 93). Recent evidence suggests that T cells appear to play more of a destructive role after SCI as Gonzalez et al. (93) demonstrated that tissue preservation and functional recovery were improved when T cell infiltration was decreased by neutralizing the chemoattractant CXCL10. In a rat model, cytotoxic T-cells were predominant compared to regulatory T cells (>90% versus 10%, respectively) further suggesting a negative role of T cells (94). In addition, secondary injury was aggravated after SCI by T cell perforin as perforin destroyed the blood spinal cord barrier triggering infiltration of inflammatory cytokines (95).

## 4 Current and future therapies for SCI

### 4.1 Pharmacological therapies

To date, we do not fully understand the clinical utility and efficacy of several immunotherapies and pharmacological agents following tSCI. These immunotherapies have the potential to improve outcomes in tSCI patients by modulating the immune response to injury and promoting regeneration, however, further preclinical and clinical studies are needed. Although our current understanding of the precise immunological mechanisms by which some of these therapies may potentially improve functional outcomes is limited, here we provide the state of the field and future directions of pharmacological therapies in tSCI.

### 4.2 Corticosteroids

The clinical utility of methylprednisolone in the treatment of traumatic SCI is controversial (48, 96–98). Theoretically, corticosteroids could prevent secondary damage after SCI and be beneficial by preserving the ultrastructure of the spinal cord by decreasing the injury induced, decreasing free radical catalyzed lipid peroxidation, enhancing impulse conduction and neuronal excitability, improving blood flow, reducing oxidative stress, and modulating the immune response (99, 100). Three landmark multicenter, double-blinded, randomized controlled trials, National Spinal Cord Injury Study I (NASCIS-I), NASCIS-II, and NASCIS-III were performed in 1984, 1990, and 1997 (96, 101, 102). NASCIS-I sought to assess the methylprednisolone dosage following SCI, but there was no placebo group in this trial due to the belief that methylprednisolone was beneficial, and it would be unethical to withhold methylprednisolone therapy (101). Significant debate has been centered around the results of the NASCIS-II which randomized 487 patients with acute traumatic SCI to receive an initial bolus of 30 mg/kg of methylprednisolone followed by an infusion of 5.4 mg/kg per hour for 23 hours or treatment with placebo or naloxone (48, 96–98). Although there was no significant benefit in the neurological outcomes among the 162 patients receiving methylprednisolone within 12 hours, analysis of a group of 65 of these patients that received methylprednisolone within 8 hours after SCI significantly showed improved neurological function at 6 months (96, 103). Therefore, the authors concluded that treatment with methylprednisolone within 8 hours improved recovery of neurological function after SCI (96). Advocates of this study tend to highlight the lack of high-quality evidence currently available within the literature while critics point to the potential arbitrary 8-hour time point, the magnitude of treatment effects, and the potent issues with losses to follow-up (98, 103–108). A prospective randomized clinical trial demonstrated no neurological benefits (109). Several class III medical evidence studies have demonstrated the beneficial effects of methylprednisolone therapy in SCI (96, 101, 102, 110, 111). Critics of these studies, however, suggest that these studies are limited by small sample sizes derived retrospectively from larger study populations and/or incomplete data sets (112–115). Another potential concern of using methylprednisolone in the treatment of acute SCI is the potential for harmful side effects (96, 101, 102, 110, 111, 116). More specifically, three Class I studies reported wound infection, hyperglycemia requiring insulin administration, and GI hemorrhage as statistically significant side effects (96, 101, 102, 110, 111, 117, 118).

Although the clinical utility of these trials remains controversial, the methylprednisolone treatment protocols outlined in NASCIS II and III are still used today (96, 102). Early critics of the NASCIS advocated that methylprednisolone therapy be considered “a treatment option” rather than “standard of care” (96, 97, 101, 102). In 2013, the routine administration of methylprednisolone in the management of acute traumatic SCI was not recommended by “Guidelines for the Management of Acute Cervical Spine and Spinal Cord Injuries” (97, 119–121). The use of methylprednisolone among surgeons has decreased based on studies surveying the practice habits of surgeons, a 2018 study suggested that a majority of surgeons worldwide (52.9%) still use methylprednisolone in the treatment of acute SCI (122–127). In the future, further studies assessing the effect of steroid therapy on the neurological outcomes of SCI patients will be necessary in order to determine its clinical utility.

### 4.3 Cyclooxygenase inhibitors

The use of non-steroidal anti-inflammatory drugs (NSAIDs) following acute SCI injury appears promising as NSAIDs have been suggested to decrease spinal cord inflammation and edema, improve motor function with minimal side effects, and increase axonal sprouting (128). The effectiveness of NSAIDs in acute SCI is likely due to their ability to inhibit Rho-A (128). In animal models after traumatic SCI, two non-steroidal anti-inflammatory drugs that can be utilized in order to maintain spinal cord blood flow are ibuprofen and meclofenamate (129, 130). Similar results have been reported with the use of both a prostacyclin analogue and a thromboxane inhibitor (131, 132). Other studies have suggested that COX-2 expression is significantly increased within the damaged rat spinal cord tissue after contusion injury, and inhibition of COX-2 enhances functional outcomes (129, 133). Small sample size and heterogeneity creates problems with studies, and the therapeutic effects seen in animals may not be the same in patients (128, 131). Given the potential for improved outcomes and the low risk for adverse side effects, well-designed prospective studies evaluating ibuprofen and indomethacin are needed in the future.

### 4.4 Minocycline

Minocycline is a chemically modified second generation tetracycline antibiotic that has the ability to penetrate the blood brain barrier (134, 135). Minocycline has been shown to provide neuroprotective effects and has been shown to have anti-inflammatory, antioxidant, and anti-apoptotic properties (131, 134, 136). In animal models of SCI, TBI, and cerebral ischemia, minocycline has been shown to protect white matter structures, motor neurons, and oligodendrocytes (137, 138). Studies suggest that minocycline following SCI had significant anti-inflammatory and anti-apoptotic effects by preventing the activation of TNFα, IL-1β, COX-2, and MMPs decreasing caspase-1 and caspase-3 levels (131, 139, 140). Minocycline has also been reported to alleviate excitotoxicity and has been suggested to inhibit the p38 mitogen activated protein kinase pathway in microglial cells (137, 141, 142).

Tissue sparing, reduction of the lesion, inhibiting oligodendroglia and neuronal apoptosis, reducing inflammation, and enhancing neurological and histological outcomes could possibly be achieved with minocycline treatment (131, 143). Studies in mice have suggested that minocycline therapy is associated with substantial improvements in long term functional outcomes in which administration of minocycline over a 4 week recovery time course increased Beattie, Basso, and Bresnahan scores at 20 days following injury (144). A 2008 study in rats is not in agreement with other preclinical and clinical studies such as Pinzon et al. (143, 145) which found that minocycline therapy did not generate tissue sparing or improved behavioral outcomes (131, 143). In clinical studies, minocycline treatment improves outcomes in acute incomplete cervical SCI patients (131, 135). A randomized, double bind, placebo-controlled, single center phase II clinical trial examined treatment with IV minocycline (n=27) for 7 days versus placebo (n=25) treatment in patients with SCI. This study showed that minocycline therapy was safe and feasible. In addition, there was a trend toward improvements in motor scores especially in tetraplegic patients. Currently, a multicenter phase III randomized controlled trial (NCT01828203) is assessing the impact minocycline therapy versus placebo therapy on acute (<12 hours) SCI patient recovery at 3 months and one year after injury. Minocycline therapy, therefore, appears to be promising for SCI, as it works *via* several mechanisms; however, more well-designed trials are necessary to definitively conclude the role of minocycline in SCI management.

### 4.5 Chondroitinase ABC enzyme

The axons of patients with SCI do not regenerate appropriately (74, 89, 131, 146). Glial reaction occurs at the site of injury, resulting in formation of a glial scar (74, 89, 131, 146). Furthermore, microglia, oligodendrocytes, and their precursors (which include astrocytes, meningeal cells, and myelin fragments) are recruited due to the glial reaction (14, 69, 89, 131, 146). Many of these cells release factors that halt axonal regeneration (74, 89, 131, 146). Chondroitin sulfate proteoglycans (CSPG) are one major class of axonal growth inhibitors that may play a key role in regeneration failure (131, 146–148). Importantly, chondroitinase ABC (ChABC) is an enzyme that could be critical in overcoming CSPG mediated inbition as ChABC digests glycosaminoglycans on CSPGs (146–149).

Animal studies have demonstrated that treatment with ChABC after SCI can dramatically improve functional recovery and regeneration of both sensory axons and the corticospinal cord (131, 150, 151). Mechanistically, ChABC most likely results in growth promoting effects through causing increased germination of spare axons, formation of new synaptic connections beneath the sites of injury, and removing perineuronal nets (152). In addition to these mechanisms, ChABC also has been shown to have immunoregulatory activity (153). More specifically, Didangelos et al. showed that ChABC increased expression of the anti-inflammatory cytokine IL-10 and reduced expression of the pro-inflammatory cytokine IL-12B (153).

In rat models, administration of ChABC promoted both improved recovery of proprioceptive and locomotor behaviors (151). In addition, below the lesion treatment with ChABC resulted in restoration of postsynaptic activity following electrical stimulation of corticospinal neurons (151). The benefits of ChABC for SCI have been clearly demonstrated in rodent models of: (a) SCI, (b) stroke, and (c) nigrostriatal injury as well as in cats with SCI (131, 154–156). A recent study in dogs with severe chronic SCI strongly supported a beneficial effect of intraspinal injection of chABC (157). Importantly, there was no evidence of long-term adverse effects with this therapy (157). Given the impressive improvements of functional outcomes with ChABC treatment and the lack of reported harmful effects, human SCI ChABC trials should be conducted in the future.

### 4.6 Monosialotetrahexosylganglioside (GM-1)

Glycosphingolipids exert several critical functions in cellular differentiation, interaction and immune response (158). Theoretically, the benefit of glycosphingolipid GM-1 stems from the ability of GM-1 to stimulate tyrosine kinase receptors, resulting in improved regeneration and enhanced neuronal plasticity (100, 131). A randomized, double blind, prospective phase II trial demonstrated significant improvement of 1-year ASIA motor scores in patients that were treated with daily GM-1 ganglioside for 18-32 days following SCI (135, 159). A randomized, double-blind, sequential multicenter phase III clinical trial examined the effect of two doses of Sygen (monosialotetrahexosylganglioside GM1 sodium salt) compared to placebo in 797 patients with acute SCI (160). Although there appeared to be accelerated bladder, bowel, and motor recovery following Sygen treatment within the first three months, ultimately there was no significant effects after the study ended (135, 160). The time of administration of GM-1 ganglioside has been postulated to be a key factor in the observed variation among the two trials (135). Furthermore, a Cochrane Database Systematic Review described significant weaknesses in data collection and presentation methodologies in these trials (135, 161). This review concluded that there is no available evidence to support treatment with gangliosides to reduce the death rate or improve the quality of life in SCI and to date no follow-up studies have been performed (161).

### 4.7 Neuroimmunophilin ligands

Neuroimmunophilin ligands are a class of compounds that have great potential for the treatment for SCI. To date, however, the results of neuroimmunophilin ligand therapy for SCI have been conflicting. Cyclosporin A and FK-506, which act as neuroimmunophilin ligands, have shown protective properties in ischemia, neurodegenerative disorders, and in trauma (131, 162, 163). In neuronal tissues, NIL-A, which is currently in Phase II clinical trials for SCI, binds with FK506 binding protein (FKBP)-12 (131, 164). Neurophilin ligand V10367 binds to FK-506 binding protein and was investigated in a preclinical model (131, 165). V10367 resulted in increased neuroregeneration in the CNS and peripheral nervous system (165). Tacrolimus in addition to its immunosuppressive effects also has neurotrophic activity and has been shown to enhance nerve regeneration in peripheral nerve injury (166, 167). Due to the potential of neuroimmunophilin ligands in the treatment of nerve injury, further preclinical studies are needed to clarify their potential role in the treatment of tSCI.

### 4.8 Anti-Nogo-A antibodies (ATI-355)

Nogo-A is neuritite growth inhibitory myelin protein which plays a key role in inhibition of neurite outgrowth and limits neurological recovery (146, 168–170). Treatment with anti-Nogo-A antibody after SCI in rat models has been shown to significantly improve recovery of locomotor training and results in superior spinal cord reorganization and regeneration (171, 172). A macaque model showed similar results (131, 173). Another study examined the therapeutic outcomes of anti-Nogo-A antibody therapy on the corticospinal tract after acute, 1 week, or 2 week delayed intrathecal anti-Nogo-A antibody infusions (131, 174). In this study, Gonzenbach et al. (174) find that treatment of SCI with anti-Nogo antibodies in rodents is limited to less than 2 weeks. A multicenter Phase I open-label cohort study assessed intrathecal administration of the human anti-Nogo-A antibody ATI355 in acute, complete traumatic paraplegia and tetraplegia (175). Administration of anti-Nogo antibodies was well tolerated and in 7 of 19 patients with tetraplegia conversion from complete to incomplete SCI injury occurred (175).

### 4.9 VX-210 (Cethrin)

The Rho signaling pathway plays a key role in neuronal growth inhibition as well as regulating the cytoskeleton and motility (176). C3 transferase, an enzyme from *Clostridium botulinum*, inhibits Rho signaling by locking RhoA in an inactive state (176). In animal models of SCI, local application of C3 transferase induced long distance cortico-spinal regeneration and improved functional outcomes (176, 177). C3 transferase therapy with wild-type C3 transferse was limited, however, by its very low levels of cell penetration (177, 178). Thus, a recombinant version that readily crosses the dura was developed BA-210 (Cethrin) (177). In addition to promoting axonal regeneration and neuroprotection, Cethrin also appears to modify the adverse immune reaction following SCI (177–179). A multicenter phase I/IIa clinical trial involving cervical and thoracic SCI patients taking 0.3 mg to 9 mg BA-210 (later VX-210) showed that BA-210 treatment significantly improved motor recovery (178, 179). More specifically, the largest changes in motor recovery were observed in cervical injury patients taking 3 mg BA-210 in which 31% of cervical injury patients, 66% of patients taking 3mg BA-210,and 6% of thoracic injury patients converted from ASIA A to AISA C or D (178, 179). A phase Iib/III study assessing whether application of VX-210 to the overlying dura would improve motor outcomes following cervical SCI was stopped prematurely as the primary efficacy end-point was not met (180).

### 4.10 Anti-CD11d Antibodies

The CD11/CD18 family of integrins are localized on the surface of leukocytes and have been shown to bind both human intercellular adhesion molecule-3 (ICAM-3) and human/rat vascular cell adhesion molecule-1 (VCAM-1) (181–184). Following injury, damaged endothelial cells upregulate VCAM-1 and ICAM-3 which facilitates leukocyte binding and migration to the site of injury (184, 185). Preclinical studies have demonstrated the promising potential of a monoclonal antibody, 217L, which binds to the CD11d subunit of the CD11/CD18 integrins (184). Initial preclinical studies in rats demonstrated that anti-CD11d therapy significantly decreased number of both neutrophils and macrophages within the cord following SCI (186, 187). Furthermore, anti-CD11d therapy substantially decreased secondary damage and resulted in superior autonomic and locomotor recovery as well as decreased neuropathic pain (188–191). Similarly, other studies have also suggested that decreasing the number of neutrophils is neuroprotective (29, 46, 85, 192–194). A CD11d monoclonal antibody study in mice, however, reported that depletion of neutrophils after SCI through the use of Ly-6G (RB6-8C5) mAb impeded rather than enhanced functional recovery (195). A follow-up study examining the therapeutic effectiveness of anti-CD11d treatment in mice demonstrated that neutrophil infiltration was reduced by 61% at 72 hours after SCI and significantly improved functional outcomes and decreased secondary damage (184).

In addition to the role of anti-CD11d therapy in decreasing the infiltration of neutrophils, studies have suggested that anti-CD11d therapy improves neurological outcomes, provides neuroprotective effects decreases pain, significantly reduces histopathological damage, and improves intraspinal serotonergic innervation patterns (190, 196). A recent study also suggested that anti-CD11d therapy decreases secondary damage by significantly reducing free radical formation (197). Given the promising results from preclinical studies, future clinical trials assessing the efficacy of anti-CD11d in humans are needed. Importantly, the mechanism of injury and injury severity may be key determinants of the efficacy of anti-CD11d therapy following SCI (198). More specifically, using a rat model Geremia et al. (198) suggested that anti-CD11d therapy is most efficacious in cases of modest injury severity with minimal non-penetrating injury and frank hemorrhage into the spinal cord (198). Thus, clinical studies assessing efficacy of anti-CD11d therapy with careful consideration of the injury severity and mechanism of injury are necessary in the future.

### 4.11 B-Cell depletion therapies

Following SCI, a robust B cell response results in the production of pathogenic antibodies that impedes neurological recovery (199, 200). The role of B cells in the pathogenesis of SCI is further supported by the fact that functional outcomes are improved in B cell deficient RAG knockout mice following SCI (201). Based on this principle, selective B-cell depletion may be neuroprotective following SCI (201, 202). Casili et al. (202) assessed the effects of antibody mediated B cell depletion in mice using a glycoengineered anti-muCD20 antibody (18B12). Casili et al. (202) demonstrated that antibody mediated B cell depletion in mice improved functional recovery, slowed neuronal death and hindlimb motor dysfunction, and substantially inhibited the NFkB-dependent production of pro-inflammatory molecules. In the future, clinical studies are necessary in order to assess efficacy of anti-CD20 antibodies including rituximab or obinutuzumab after SCI (202, 203).

### 4.12 Targeting T-cells: trafficking, infiltration, and depletion

After SCI, T cells can directly or indirectly effect neurons, glia, or other CNS cells through the production of tumor necrosis factor (TNF)-α, interleukins (ILs), or other proinflammatory cytokines that play key roles in cytotoxic cell damage (92, 202, 204). Studies have demonstrated that therapeutic strategies targeting T cell infiltration and trafficking are neuroprotective (205). More specifically, antagonism or neutralizing antibodies against CXCL10, a known T cell recruiter, has been shown to improve functional outcomes, decrease T cell infiltration, decrease neuronal death, and significantly increase regeneration of axons (8, 206, 207). Another promising immunosuppressive therapy following SCI, involves the use of fingolimod, which targets sphingosine 1-phosphate receptor (S1P1), induces internalization of S1P1, decreases the number of circulating lymphocytes, and prevents trafficking of lymphocytes into tissues by sequestering lymphocytes within lymph nodes (208, 209). Locally administered fingolimod has been shown to result in superior functional recovery, decreases reactive gliosis, and prevents neuronal cell death (203, 210). Systemic treatment of fingolimod after SCI has been shown to prevent neuroinflammation and significantly improve both bladder and motor function (211, 212). Given the promising results of T cell targeting therapies, further clinical studies are necessary in order to assess the efficacy of CXCL10 antagonisms and fingolimod after SCI.

### 4.13 Macrophage transplantation

Previously, it has been shown that the macrophage immune response to injury was both delayed and blunted in the CNS of mature rodents compared to the more regenerative peripheral nervous system (213–215). Rapalino et al. (216) showed that local injection of homologous macrophages activated *ex vivo* through incubation with homologous peripheral nerves triggered partial motor recovery in rats with complete spinal cord transection (216, 217). Incubation of macrophages with autologous skin has been shown to result in equivalent effects and is thought to produce an “alternatively activated wound-healing phenotype” (213, 218). More specifically, in rats with a contused spinal cord, injection of macrophages activated with skin resulted in decreased spinal cyst formation and enhanced motor recovery (213, 218). This study also demonstrated that the skin activated macrophages had increased levels of cell surface markers that are characteristic of antigen presenting cells and macrophage secretion of brain-derived neurotrophic factor and interleukin-1b (213, 218). Autologous macrophage therapy has exciting potential due to the key functions macrophages play including clearing tissue debris from the site of injury, ability to modulate the immune system and influence spinal cord neurons, glial cells, and immune cells (217, 218). Safety studies in animals reported no short-or long-term adverse effects or toxicity in animals treated with macrophages to the Food and Drug Administration under Investigational New drug application No. 8427 (217).

Based on preclinical studies, an open-label phase I clinical trial assessing the safety and tolerability of treatment with incubated autologous macrophages in acute complete SCI patients was conducted (217). In this trial 3 of 8 subjects demonstrated conversion from AIS A to AIS C and overall the therapy was well tolerated (217). A single-blinded primary outcome randomized controlled trial involving six treatment centers in the United States and Israel, however, did not demonstrate a significant difference in the primary outcome among the autologous incubated macrophage treatment group (n=26) and the control group (n=16) (213). In the future, additional studies and further protocol optimization will be necessary to assess the efficacy of treatment of SCI with autologous incubated macrophages.

### 4.14 Hepatocyte growth factor

Hepatocyte growth factor (HGF) binds to c-Met and plays key roles in protection, regeneration, homeostasis, and exerts anti-inflammatory effects in several organs (219). Studies in rodent models have suggested that treatment with HGF results in the neuroprotective effects, enhanced angiogenesis, reduction of the lesion size, improved neuronal survival, and a decrease in production of oligodendrocytes (131, 220, 221). Furthermore, injection of HGF expression vector into rat spinal cords resulted in higher rates of survival, regeneration of axons and oligodendrocytes, and increased angiogenesis (131, 222). In a primate model of cervical SCI, significant improvements in hand dexterity were seen after HGF treatment (221, 223). Another study showed that exogenous treatment of HGF decreased astrocyte activation, decreased glial scar formation, decreased leukocyte infiltration, and produced anti-inflammatory effects (222). A recent double blind, randomized phase I/II clinical trial demonstrated the safety of treatment with recombinant human HGF and a larger phase III trial is necessary in the future in order to assess efficacy (224).

### 4.15 Neurotrophic factors

Neurotrophic factors are molecules that control the growth, survival, proliferation, and differentiation of neurons (225). In addition, neurotrophic factors play key roles in modulating immune responses and autoimmunity (226). Neurotrophic factors are often delivered with the use of collagenases (152). Delivering neurotrophic factors *via* collagenases has been reported to be more effective compared to delivery *via* direct injection, growth factor-saturated gel, or continuous injection (131, 152, 227). *Ex vivo* therapy can also be used when a patient’s cells are removed, genetically modified to synthesize specific neurotrophic factors, expanded in culture, and retransplanted into the patient (131, 152). The benefit of this route is that it provides localized, high-dose growth factors on delivery; however, initial studies suggest that *ex vivo* therapy is not ideal at stimulating distal axonal growth following initial axon growth (227).

### 4.16 Fibroblast growth factors

Fibroblast growth factors are a family of growth factors that are present in both the central and peripheral nervous system that play key roles in determining cell fate, differentiation, migration, and display neuroprotective effects (228–230). In addition to their neuroprotective effects, FGFs also may have some immunomodulatory effects as one approach utilizing acidic FGF (aFGF) combined with peripheral nerve grafts in rats following transection SCI increased levels of IL-4, IL-10, and IL-13 (9, 231). Other studies have shown FGFs protect against excitotoxicity and prevent the production of free radicals (131, 232). Treatment with FGF1 and FGF2 has been shown to significantly increase the growth and survival of various types of neurons including dopaminergic, cerebellar, hippocampal, and isolated sensory neuronal cells (143, 228, 233–235). Furthermore, both keratinocyte growth factor and basic FGF treatments appears to provide neuroprotection following SCI (131, 236). A prospective, open label uncontrolled clinical study demonstrated the safety, feasibility, modest improvements in functional outcomes in chronic SCI patients at 48 months that were treated with survival nerve grafts with fibrin glue containing acidic FGF (aFGF) (131, 237). Another clinical trial involving nine patients with cervical SCI treated with direct implantation of fibrin glue with aFGF for over six months showed significantly improved ASIA motor and sensory scale scores between the preoperative and follow-up (6-months postoperative) (131, 238). In addition, a prospective, open-label, uncontrolled clinical trial with 60 recruited patients with SCI demonstrated substantial improvement in AISA motor and sensory scale scores after 24 months following FGF therapy (239).

### 4.17 Granulocyte colony-stimulating factor

Granulocyte colony-stimulating factor (G-CSF) is a cytokine that in addition to its role in inducing proliferation, survival, and development of granulocyte lineage cells has been shown to play key roles in functional recovery following neuronal injury (240, 241). More specifically, studies have shown that G-CSF decreases both neuron and oligodendrocyte apoptosis, suppresses inflammatory cytokines (reduces TNFα and IL-1β in the spinal cord lesion), inhibits glutamate excitotoxicity, enhances angiogenesis, and promotes proliferation/mobilization of neuronal cells (131, 242–245). Based on these studies, phase I/IIa clinical trials were conducted and confirmed both the feasibility and safety of G-CSF therapy following SCI (246–248). The sentence should now read: "A phase II multicenter, prospective, non-blinded, nonrandomized clinical trial assessing patients with acute cervical SCI (within 48 hours after injury) receiving G-CSF therapy versus control therapy showed significant improvement (P<0.01) of motor paralysis and ASIA scores even at 1 year after injury in patients receiving G-CSF therapy compared to patients receiving control therapy (249). A recent phase 3 prospective, randomized, double-blind, and placebo controlled comparative trial was conducted and failed to show significant changes in ASIA motor scores from baseline to three months after G-CSF therapy (245). Additional randomized controlled trials will be needed in the future in order to determine the therapeutic benefits of SCI. Immunological pharmaceutical clinical studies are summarized in **Table 1**.

**Table 1:** Immunological pharmaceutical clinical studies summary table.

Year	First author	Study Type	Conclusion	Treatment
1984	Bracken	Multicenter double-blind randomized trial	No difference in neurological recovery of motor function or pinprick and light touch sensation was observed between the two treatment groups six weeks and six months after injury.	Corticosteroids
1990	Bracken	Multicenter randomized, double-blind, placebo-controlled trial	In patients with acute spinal-cord injury, treatment with methylprednisolone in the dose used in this study improved neurologic recovery when the medication was given in the first eight hours.	Corticosteroids
1993	Galandiuk	Prospective and retrospective	These data do not permit a judgment to be made whether neurologic status was improved by S administration. It is known that vital immune responses were adversely affected, that pneumonia was somewhat more prevalent, and that hospitalization was prolonged and costs therefore increased by an average of $51,504 per admission.	Corticosteroids
1993	Kiwerski	Prospective, controlled	Greater improvement when treated with dexamethasone, increased risk gastrointestinal bleeding and delayed wound healing.	Corticosteroids
1994	Shepard	Multicenter, randomized	No evidence of compromised liver function from this steroid protocol.	Corticosteroids
1997	Bracken	Double-blind, randomized clinical trial	Patients treated with tirilazad for 48 hours showed motor recovery rates equivalent to patients who received methylprednisolone for 24 hours.	Corticosteroids
1997	Gerndt	Retrospective review with historical control	Although the NASCIS-2 protocol may promote early infectious complications, it had no adverse impact on long-term outcome in patients with ASCIs.	Corticosteroids
1998	Petitjean	Prospective, randomized clinical trial	No neurologic benefit from treatment was observed.	Corticosteroids
1998	Wing	Prospective cohort study	The true incidence of AVN among the methylprednisolone treated group is less than 5% and therefore they continue to recommend short term (24 h) methylprednisolone therapy.	Corticosteroids
2001	Matsumoto	Prospective, randomized, and double-blind study	Aged patients with cervical spinal injury may be more likely to have pulmonary side effects (P = 0.029) after high-dose therapy with MPSS.	Corticosteroids
2003	Pollard	Retrospective study	In traumatic, incomplete, cervical spinal cord injuries, neurologic recovery was not related to high-dose methylprednisolone administration.	Corticosteroids
2006	Tsutsumi	Retrospective, single center	Improved motor scores with MPSS treatment in those with incomplete paralysis at admission but not with those with complete paralysis.	Corticosteroids
2007	Lee	Retrospective	In patients with cervical spinal injury secondary to blunt injuries, treatment with MP improves motor/sensory function, but harmful side effects limit its functional efficacy in patients with complete ASCI.	Corticosteroids
2015	Evaniew	Propensity score-matched cohort study from a Canadian multi-center spinal cord injury registry	Methylprednisolone did not improve motor recovery in acute TSCIs, and there was a higher rate of total complications in the methylprednisolone group.	Corticosteroids
2012	Casha	Phase II	In acute spinal cord injury, minocycline treatment is feasible, safe, and associated with a tendency towards improvement.	Minocycline
1991	Geisler	Prospective, randomized, placebo-controlled, double-blind trial	GM-1 enhanced the recovery of neurologic function after one year.	GM-1
2001	Geisler	Randomized, double-blind, sequential, multicenter clinical trial of two doses of Sygen^®^versus placebo	Primary efficacy analysis showed a trend but did not reach significance.	GM-1
2018	Kucher	Phase I	ATI335 was well tolerated in humans; efficacy trials using intrathecal antibody administration may be considered in acute SCI.	Anti-Nogo-A
2011	Fehlings	Phase I/IIa	The observed motor recovery suggested that BA-210 may increase neurological recovery after complete SCI.	VX-210 (Cethrin)
2013	McKerracher	Phase I/IIa	Time to recruitment was examined, and it was found that the extent of motor and sensory recovery could not be explained by early surgery, and the Cethrin-treated patients showed favorable trends compared with the Surgical Timing in Acute SCI data.	VX-210 (Cethrin)
2021	Fehlings	Phase IIb/III	The primary efficacy end-point was not met, with no statistically significant difference in change from baseline in upper-extremity motor score at 6 months after treatment between the VX-210 (9-mg) and placebo groups.	VX-210 (Cethrin)
2005	Knoller	Phase I	Incubated autologous macrophage cell therapy is well tolerated in patients with acute SCI.	Macrophage Transplant
2012	Lammertse	Phase II	The analysis failed to show a significant difference in primary outcome between the two groups. The study results did not support treatment of acute complete SCI with autologous incubated macrophage therapy as specified in the protocol.	Macrophage Transplant
2020	Nagoshi	Phase I/II	Subjects did not show any serious adverse events caused by KP-100. KP-100 has the potential to be useful and beneficial for SCI patients during the acute phase.	HGF
2008	Wu	Phase I	Modest nerve regeneration occurred in all 9 patients after procedure without any observed adverse effects.	FGF
2011	Wu	open-label, prospective, uncontrolled human clinical trial	FGF safe/feasible; improvement in motor/sensory scores, ASIA impairment scales, neurological levels, and functional independence.	FGF
2018	Ko	Phase III	aFGF was safe, feasible, and could yield modest functional improvement in chronic SCI patients.	FGF
2012	Takahashi	Phase I/IIa	G-CSF is safe and some neurological recovery may occur in most patients.	G-CSF
2014	Inada	Phase I/IIa	G-CSF has beneficial effects on neurological recovery in patients with acute SCI.	G-CSF
2014	Saberi	Phase I/II	G-CSF administration in motor-incomplete SCIs was associated with significantly higher motor improvement, and also the higher the initial ASIA Impairment Scale (AIS) grade, the less would be the final AIS change.	G-CSF
2015	Kamiya	Phase I/IIa	G-CSF administration is safe and effective.	G-CSF
2018	Derakhshanrad	Phase III	Administration of G-CSF for incomplete chronic spinal cord injuries was associated with significant motor, sensory, and functional improvement.	G-CSF
2021	Koda	Phase III	Failed to show a significant effect of G-CSF in primary end point but sub-analyses suggested potential G-CSF benefits for a specific population.	G-CSF

## 5 Stem cell therapy for SCI: Recent developments, limitations, and future directions

Stem cell therapy has potential to be revolutionary in the management of SCI as stem cell therapy can possibly regenerate neurological networks, modulate neuroinflammation, and ameliorate damage. Although many transplanted stem cell clinical trials for traumatic SCI patients are in the early stages (phase 1/2), there recently have been some promising results (250). Several studies have demonstrated safety and early evidence of potential efficacy of transplanted mesenchymal stem cells (MSCs), human neural stem/progenitor cells (hNSPC) autologous human Schwann cell (ahSCs), human umbilical cord-derived Wharton jelly mesenchymal stromal cells (WJ-MSCs), and autologous bone marrow derived mononuclear cells administered through multiple different routes (251–273). Transplanted stems cells exert their effects *via* three distinct mechanisms including: (1) cell replacement (274–276), characterized by the differentiation of stem cells into neuronal or vascular cells to compensate for the decreased function due to injury; (2) stem cell regeneration (31, 277, 278), in which transplantation of stem cells triggers the regeneration of the patient’s neuronal stem cells; and (3) functional multipotency (279), in which transplanted stem cells secrete a multitude of trophic factors which ameliorate damage to the nervous system or assists in the regeneration of new neuronal circuits. Interestingly, Kishk et al. suggested a potential contraindication of autologous MSCs in patients with a past medical history of myelitis (280).

Currently, the majority of clinical trials have focused on stem cell transplant in severely injured SCI patients (ASIA A) that are >6 months after SCI (chronic stage) (250). To date, there is minimal hope for spontaneous recovery, and there are no effective treatments for chronic stage (>6 months following injury) ASIA A patients, which makes the potential benefits of stem cell transplantation an attractive option (250). The results of trials of stem cell transplantation in chronic stage SCI patients have varied with some trials demonstrating recovery rates as high as 100% while other trials have shown no improvement based on ASIA impairment scale (256, 281, 282). Within the studies that suggested no improvement based on AISA impairment scale, patients did show some improvement on somatosensory evoked potential (SEP) and motor-evoked potential (MEP) testing suggesting some benefit in these patients (250, 281, 282). Other studies have suggested higher rates of SCI recovery after receiving stem cell therapy in matched-control patients (283, 284). In a group of 70 patients randomly divided, 34% of patients that were treated with intrathecal bone-marrow-derived mesenchymal stromal cell therapy demonstrated improvement in AISA grade compared to 0% of patients in the control group (250, 285). Similarly, in a group of 34 ASIA A SCI that were randomly divided into a control group, rehabilitation group, and a cell transplantation group, only patients that received cell transplantation showed significant motor, sensory, and urinary recovery compared to their status prior to treatment (250, 286).

Although a majority of preclinical animal studies have focused on delivering stem cell therapy within the acute phase after SCI (within the first 24 hours), there are relatively few clinical trials that examine stem cell therapy within the first 24 hours after SCI (287). Due to the unforeseen requirement for stem cells such as human umbilical cord mesenchymal stromal cells (MSCs) for stem cell therapy following traumatic SCI, it is unfortunately not feasible to use autologous material as the cells would first need to be expanded ex vivo (288). Thus, stem cell therapy during the acute phase of traumatic SCI requires the use of allogenic material. Two complete injury ASIA A patients that were treated with allogenic human umbilical cord mesenchymal stromal cells that were transplanted using a collagen scaffold showed functional recovery to an incomplete injury (ASIA C) (289). More trials in the future will be necessary to assess the potential benefits of acute phase stem cell transplant in traumatic SCI patients. One potential confounder to consider in the acute phase when assessing the efficacy of stem cell therapy is that within the acute phase, spontaneous recover is possible (250).

Finally, some trials and studies have assessed stem cell therapy administered between 2 days and 6 months following SCI, which has been defined as the sub-acute phase (250). The results of stem cell therapy trials within the sub-acute period of SCI have produced variable results with some trials demonstrating significant improvement and another group of trials suggesting no significant recovery (264, 281, 290, 291). One study examined the effectiveness of intra-spinal bone-marrow-derived mononuclear cells in patients with complete SCI (292). This study showed that when treated within 8 weeks following SCI, 30% of patients had significant functional recovery compared to 0% when stem cell transplantation was performed at >8 weeks and 7.6% AISA improvement in matched control patients (250, 292). A different study reported that significant improvement within the stem cell therapy group compared to the control group in which 46% of AISA A patients improved to ASIA C following injection of intrathecal bone-marrow-derived mesenchymal stromal cells (BMSC) versus 15% in the control group (290).

### 5.1 Future of stem cell therapy in SCI

Clinical trials, preclinical studies, and meta-analyses have shown that although the efficacy of stem cell therapy is promising, many challenges remain which will require further optimization and innovative solutions (293). One of the biggest and most concerning challenges of stem cell therapy for spinal cord injury moving forward is the potential for adverse effects. Although many clinical trials have been reported as safe without mortalities or severe morbidity, tumorigenicity, immunogenicity, and cell therapy-related immunotoxicity are potentially serious consequences of stem cell therapy (294). More specifically, to date there have been at least 28 kinds of adverse effects reported (293). Some side effects reported are likely secondary to the delivery procedures rather than the cells (294). Side effects secondary to the delivery procedure itself likely include subarachnoid hemorrhage, cerebrospinal fluid leaks, and transient deterioration in sensorimotor symptoms (25, 217, 280, 294–297). Moving forward, innovative delivery strategies will need to be investigated and employed to reduce side effects and further improve efficacy. In addition, side effects not associated with the transplanted cells and/or procedures were reported and include but are not limited to vomiting, pulmonary thromboembolism, fever, body aches, urinary tract infection (217, 262, 273, 291, 292, 294, 296, 298). Unfortunately, comprehensive assessment of safety and efficacy of stem cell therapy has been challenging for SCI patients as clinical trials in the past have been limited by relatively low sample sizes and insufficient control groups (299). In the future, large preclinical studies and early clinical studies are necessary in order to assess the safety of transplantation of human stem cells. Without rigorous assessment of the safety and potential adverse effects, the widespread adaptation of stem cell therapy will continue to be hampered.

Next, the properties and characteristics of the stem cells themselves must be rigorously assessed in order to continue to improve efficacy. Arguably the most critical factor in response to stem cell therapy is the origin of the stem cells used in the therapy (269, 300, 301). In addition to the studies examining MSCs, studies have also suggested potential feasibility and safety of human neural stem cell transplantation, but the clinical efficacy of such therapies requires further study. Similarly, studies examining autologous human Schwann cell transplantation have demonstrated feasibility, although currently they show unclear benefit (296, 302). Multiple studies have also demonstrated safety and feasibility of olfactory mucosa autografts (297, 303). To date, factors such as cell survival and integration are hurdles that potentially explain some of the heterogenous results among clinical trials must be improved through more carefully standardizing experimental designs.

The dose of stem cells also appears to be an especially critical factor and has varied significantly among trials from 10^6^ to 10^10^ cells (250, 304). The route of administration of stem cells also appears to have a significant impact on therapy as intraspinal approaches result in the highest level of cell engraftment but is highly invasive compared to intravenous approaches which have been suggested to result in the lowest number of cells within the damaged lesion (250, 264). Furthermore, the optimal timing of stem cell transplantation in SCI remains to be determined. One pitfall to stem cell therapy moving forward is that a majority of clinical trials are single-centered investigator-oriented trials and therefore, further standardization including standardization between agencies will be necessary moving forward (250). Stem cell clinical studies are summarized in **Table 2**. In the future, clear and standardized transplantation strategies, cell numbers, treatment times, storage protocols, and consistent generation protocols which produce genetically stable and consistent therapeutic cells will be critical to unlock the potential efficacy of stem cell therapy for spinal cord injury

**Table 2:** Stem cells clinical studies summary table.

Year	First Author	Study Type	Conclusion	Treatment
2005	Feron	Phase I	Transplantation of autologous olfactory ensheathing cells into the injured spinal cord is feasible and is safe.	Stem Cell
2006	Lima	Prospective, case series	Olfactory mucosa autograft transplantation into the human injured spinal cord was feasible, relatively safe, and potentially beneficial.	Stem Cell
2006	Moviglia	Prospective, case series	Minimally invasive administration of AT-NSC showed minor adverse events, and was effective for the repair of chronic spinal cord lesions.	Stem Cell
2006	Sykova	Phase I/II	Autologous bone marrow cells appears to be safe	Stem Cell
2007	Chernykh	Prospective, controlled	Transplantation of autologous bone marrow cells can be a novel safe strategy for the treatment of patients in the late period after spinal trauma.	Stem Cell
2007	Yoon	Phase I/II	BMC transplantation and GM-CSF administration were not associated with any serious adverse clinical events increasing morbidities. The AIS grade increased in 30.4% of the acute and subacute treated patients (AIS A to B or C), whereas no significant improvement was observed in the chronic treatment group.	Stem Cell
2008	Deda	Prospective, case series	BM-derived autologous stem cell therapy was effective and safe for the treatment of chronic SCI.	Stem Cell
2008	Geffner	Pilot clinical study, prospective	These studies demonstrate that BMSCs administration *via* multiple routes was feasible, safe, and may improve the quality of life for patients living with SCI.	Stem Cell
2008	Mackay-Sim	Phase I/IIa	Transplantation of autologous olfactory ensheathing cells into the injured spinal cord was feasible and safe up to 3 years of post-implantation.	Stem Cell
2008	Saberi	Prospective, case series	Autologous Schwann cell transplantation was generally safe for the selected number of SCI patients, but it did not prove beneficial.	Stem Cell
2009	Cristante	Prospective	Peripheral blood stem cells were safe and improved SSEPs in patients with complete SCI.	Stem Cell
2009	Kumar	Phase I/II	Transplant of autologous human bone marrow derived mononuclear cells through a lumbar puncture was safe, and one-third of spinal cord injury patients showed perceptible improvements in the neurologic status.	Stem Cell
2009	Pal	Pilot clinical study, prospective	Safety was observed with no serious adverse events following transplantation of BM MSCs in SCI patients.	Stem Cell
2010	Kishk	Case control series	Autologus MSCs may have side effects and may be contraindicated in patients with a history of myelitis.	Stem Cell
2010	Lima	Phase I/II	OMA was feasible, relatively safe, and possibly beneficial in people with chronic SCI when combined with postoperative rehabilitation.	Stem Cell
2011	Bhanot	Pilot clinical study, prospective	Though the administration of allogenic human mesenchymal stem cells was safe in patients with SCI, it may not be efficacious; especially in patients with chronic SCI.	Stem Cell
2011	Ra	Phase I	The systemic transplantation of hAdMSCs appeared to be safe and did not induce tumor development.	Stem Cell
2012	Frolov	Prospective, sex/age matched control	The local effects of autologous hematopoietic stem cell treatment at the cervical level were evaluated by median SEP and wrist muscle MEP demonstrated the ability of stem cells to spread within the spinal cord at least from lumbar to the cervical level, home there, and participate in the neuro-restoration processes.	Stem Cell
2012	Karamouzian	Phase I/II	Transplantation of autologous BMC *via* LP was a feasible and safe technique.	Stem Cell
2012	Park	Prospective, case series	Three of the 10 patients with SCI who were directly injected with autologous MSCs showed improvement in the motor power of the upper extremities and in activities of daily living, as well as significant magnetic resonance imaging and electrophysiological changes during long-term follow-up.	Stem Cell
2012	Sharma	Prospective, case series	The results showed that intrathecally and intramuscularly administered autologous bone marrow-derived mononuclear cell was safe, efficacious, and also improved the quality of life of children with incurable neurological disorders and injury.	Stem Cell
2013	Dai	Prospective, randomized, control	BMMSCs transplantation improved neurological function in patients with complete and chronic cervical SCI.	Stem Cell
2014	Al-Zoubi	Prospective, case series	This study presents a safe method for transplanting specific populations of purified autologous SCs that can be used to treat SCIs in a clinical setting.	Stem Cell
2014	Cheng	Prospective, randomized, control	UCMSC transplantation effectively improved neurological functional recovery after spinal cord injury, and its efficacy was superior to that of rehabilitation therapy and self-healing.	Stem Cell
2014	El-Kheir	Phase I/II	When combined with physical therapy, autologous adherent bone marrow cell therapy appeared to be a safe and promising therapy for patients with chronic SCI of traumatic origin.	Stem Cell
2014	Mendonca	Phase I	Intralesional transplantation of autologous mesenchymal stem cells in subjects with chronic, complete spinal cord injury was safe, feasible, and may promote neurological improvements.	Stem Cell
2015	Shin	Phase I/IIa	Transplantation of hNSPCs into cervical SCI was safe and well-tolerated and was of modest neurological benefit up to 1 year after transplants.	Stem Cell
2016	Hur	Pilot clinical study, prospective	Intrathecal transplantation of autologous ADMSCs for SCI was free of serious adverse events, and several patients showed mild improvements in neurological function.	Stem Cell
2016	Oh	Phase III	Single MSCs application to intramedullary and intradural space was safe, but had a very weak therapeutic effect compared with multiple MSCs injection.	Stem Cell
2016	Satti	Phase I	Autologous MSCs were safely administered through intrathecal injection in spinal cord injury patients.	Stem Cell
2016	Vaquero	Phase I/II	Personalized cell therapy with MSCs was safe and led to clear improvements in clinical aspects and quality of life for patients with complete and chronically established paraplegia.	Stem Cell
2017	Anderson	Phase I	It was feasible to identify eligible candidates, appropriately obtain informed consent, perform a peripheral nerve harvest to obtain SCs within 5–30 days of injury, and perform an intra-spinal transplantation of highly purified autologous SCs within 4–7 weeks of injury.	Stem Cell
2017	Vaquero	Phase II	Administration of repeated doses of MSCs by subarachnoid route was a well-tolerated procedure that was able to achieve progressive and significant improvement in the quality of life of patients suffering incomplete SCI.	Stem Cell
2018	Curtis	Phase I	The results support the safety of NSI-566 transplantation into the SCI site and early signs of potential efficacy in three of the subjects warrant further exploration of NSI-566 cells in dose escalation studies.	Stem Cell
2018	Levi	Phase II	Interim analysis of Cohorts I and II demonstrated a trend toward Upper Extremity Motor Score (UEMS) and Graded Redefined Assessment of Strength, Sensibility, and Prehension (GRASSP) motor gains in the treated participants but at a magnitude below the required clinical efficacy threshold set by the sponsor to support further development resulting in early study termination.	Stem Cell
2018	Levi	Phase I/II	A total cell dose of 20 M cells *via* 4 and up to 40 M cells *via* 8 perilesional intramedullary injections after thoracic and cervical SCI respectively proved safe and feasible using a manual injection technique.	Stem Cell
2018	Vaquero	Phase II	Autologous MSCs for SCI was safe and showed efficacy in patients with SCI, mainly in recovery of sphincter dysfunction, neuropathic pain, and sensitivity.	Stem Cell
2018	Vaquero	Phase II	MSCs could be considered as a new alternative to the treatment of post-traumatic syringomyelia, achieving reduction of syrinx and clinical improvements in individual patients.	Stem Cell
2018	Xiao	Prospective, case series	Supraspinal control of movements below the injury was regained by functional scaffolds implantation in the two patients who were judged as the complete injury with combined criteria, it suggested that functional scaffolds transplantation could serve as an effective treatment for acute complete SCI patients.	Stem Cell
2019	Levi	Phase II	There was a trend toward Upper Extremity Motor Score (UEMS) and Graded Redefined Assessment of Strength, Sensibility, and Prehension (GRASSP) motor gains in the treated participants, but at a magnitude below the required clinical efficacy threshold set by the sponsor to support further development resulting in early study termination	Stem Cell
2020	Albu	Phase 1/IIa	Intrathecal transplantation of human umbilical cord-derived WJ-MSCs was safe. A single intrathecal infusion of WJ-MSCs in patients with chronic complete SCI induced sensory improvement in the segments adjacent to the injury site.	Stem Cell
2021	Gant	Phase I	Feasibility and safety were shown for ahSC transplantation combined with a multi-modal rehabilitation protocol for participants with chronic SCI.	Stem Cell
2021	Honmou	Phase II	Feasibility, safety, and functional improvements with infused MSCs into patients with SCI was observed.	Stem Cell

Importantly, recent evidence suggests that the biological and therapeutic effects of stem cell therapy including MSCs are contained within extracellular vesicles and secreted factors (305–307). Next, we review the exciting potential of EV therapy.

## 6 Extracellular vesicle therapy for SCI: An exciting new direction

Extracellular vesicles (EVs) are cell derived, nanosized sacs that are encapsulated with a lipid bilayer and enriched for nucleic acids, lipids, and proteins (308, 309). EVs are most commonly classified based on their route of biogenesis and size in which exosomes (40-150 nm) are produced *via* the endolysosomal pathway, microvesicles (150-1000 nm) are blebbed from the cell membrane, and apoptotic bodies (1,000-5,000 nm) are released as a part of programmed cell death from the plasma membrane (14). EVs have been shown to be released from almost all cells and have been isolated from almost all body fluids (310–313). Importantly, exosomes play a crucial role in intercellular communication in diverse cellular processes including immune response (314, 315). The initiation signal for the peripheral immune response following traumatic SCI is unclear (14, 316). Both neuronal and humoral hypotheses have been investigated; however, to date no consistent molecular candidates have been identified that fully account for initiation of the peripheral immune response (14, 316–318). Recent evidence suggests that EVs may be the missing link as they mediate communicate between distant organs (14, 319). Furthermore, EVs are attractive for therapeutic purposes because accumulating evidence suggests that the potential therapeutic benefit of MSC stem cell therapy is primarily due to the secreted factors including EVs and is not due to the direct cell-cell interactions within the injured tissues (305–307). EVs also have exciting therapeutic potential due to their ability to target specific cell types and their inherently biocompatibility (319, 320). Below, we review the recent developments and outline the future directions necessary in order to apply this potentially revolutionary therapy to SCI patients.

Reports have suggested that traumatic SCI induces a significant increase in plasma-derived EVs during the acute phase of injury (14). To date, however, studies characterizing EVs after SCI are lacking compared to the number of studies investigating EVs following traumatic brain injury (TBI) (14, 319). It is important to emphasize that although TBI data may provide some insight into the role of EV signaling following SCI injury, the impact of EVs on lesion progression and the overall impact on the acute phase response likely differ in traumatic SCI compared to TBI due to factors such as: (a) anatomical differences in the distribution of gray and white matter; (b) the phenotype and distribution of microglia in the spine compared to the brain; (c) increased local CXC chemokine expression and neutrophil recruitment to the spinal cord compared to the brain; and (d) greater breakdown of the blood spinal cord barrier compared to the blood brain barrier (14, 321). One study showed that EVs isolated from deceased SCI patients had a proinflammatory phenotype as EVs were enriched for inflammasome associated proteins including caspase-1, NLRP1, and ASC (322). In 2020, two studies reported EV-associated RNA-sequencing data from the serum of rats at 1 and 7 days postinjury (323, 324). Another study showed dynamic alterations in EVs in a mouse model following a thoracic contusion SCI (325). In this study, Khan et al. (325) found that there was a robust increase in plasma tetraspanin CD81^+^ extracellular vesicles after SCI and an overall decrease in total plasma EV. A significant decrease in CD81 surface expression on astrocytes was also observed by Khan et al. (325) at the site of injury suggesting that these cells may release CD81 positive EVs into the blood.

Astrocytes had previously been suggested to regulate the acute phase response (APR) (326). Based on this evidence, plasma EVs from mice with SCI or from uninjured control mice were intracerebroventricularly injected into healthy mice to assess the role of circulating EVs in promoting inflammation in recipient target organs (319, 325). Injection of EVs from SCI mice into healthy mice resulted in increased expression of several key inflammatory genes including astrocyte reactivity markers within 24 hours after injection of SCI EVs compared to injected EVs from uninjured mice (319, 325, 327). In addition, flow cytometry showed increased intracellular IL-1β and IL-1α levels in brain astrocytes from injected SCI Evs compared to EVs from uninjured mice (319, 325, 327). Therefore, this recent data suggests that astrocytes may play a key role in the production of EVs and regulating the APR following SCI (319, 325). In order to design and optimize EV therapies for traumatic SCI, it will be imperative for future studies to focus on characterizing the circulating population of EVs in SCI and to characterize the pathophysiological functions of EVs in SCI as there may be key differences in the role of EVs in SCI versus TBI.

To date, most studies have employed EVs from mesenchymal stem cells and have consistently shown improved recovery of function and behavior deficits in SCI and TBI models (306, 328, 329). For example, Jia et al. reported that injecting sonic hedgehog (Shh)-overexpressing bone mesenchymal stem cell derived exosomes was an effective therapy in a rat SCI model as there was an improvement in hind limb motor function based on Basso-Beattie-Bresnahan scores relative to both untreated and control bone mesenchymal stem cell derived exosome treatment groups (330). In addition, Li et al. (331) reported significant nerve recovery through attenuation of inflammation and oxidation in a rat transection SCI model receiving human mesenchymal stem cell derived exosomes immobilized in an adhesive hydrogel. Interestingly, Guo et al. (332) reported that intranasal administration of mesenchymal stem cell derived exosomes could cross the blood brain barrier, travel to area of the SCI, and when loaded with phosphatase and tensin homolog small interfering RNA, these exosomes decreased astrogliosis, decreased microgliosis, improved axonal growth, and increased neovascularization.

In addition to the consistently improved recovery of function in SCI models administered EVs, EVs are attractive compared to cell-based therapies as they mitigate concerns of uncontrolled proliferation or differentiation of cellular transplants and immunogenicity (319, 320). Excitingly, EV therapy compared to administration of their parental cells appears to result in superior outcomes as a recent study demonstrated that intravenous administration of human umbilical cord mesenchymal stromal cell (hUC-MSC) derived extracellular vesicles resulted in more effective modulation of the systemic immune response and more efficiently reduced scarring and inflammation compared to administration of parental MSCs (333). Other studies have also reported markedly reduced inflammation and improved functional outcomes with MSC-derived EV administration following traumatic SCI (334–336). In addition to the role of EVs modulating the systemic immune response, the potential of a higher local concentration and more rapid contact with the tissue lesion may also facilitate improved outcomes compared to cell therapies although more studies are needed (288, 333). Interestingly, reduction of circulating neutrophils and retention of monocytes within the spleen have been suggested to result in improvement in locomotor function in a SCI model administered MSC-EVs (306). Previous studies have shown that MSC-EVs localize to the spleen, and splenectomies have been associated with improved neurological outcomes in SCI models (14, 306, 337, 338). Future studies to assess whether the benefit of MSC EVs would remain in SCI models that had undergone splenectomy are needed (14).

### 6.1 Principles of design, limitations, and future directions of EV therapy

Arguably the cell source of the EVs is the most critical factor in manufacturing EVs for therapeutic purposes as the phenotype of EVs including the cell-surface proteins, cytosolic proteins, and composition of the nucleic acids that are available to be packaged into EVs are at least partly dictated by the cell source (339–342). For instance, the EV content of one critical class of small ncRNAs and miRNAs is significantly different among cell sources suggesting the importance of cell source as an EV design criterion (343, 344).

The cell culture conditions and microenvironment are also critical in order to tune the therapeutic bioactivity of EVs (319). Parameters including cell density seeding, cell age/passage, and the collection frequency of EVs have been reported to result in significant changes in production rates and the therapeutic activities in several different cell types (319, 345). For instance, EVs isolated from stressed astrocytes that were oxygen and glucose deprived resulted in neuroprotection *in vitro* most likely due to an increase in EV associated miR-92b-3p content compared to astrocytes that were not oxygen and glucose deprived (346, 347). The microenvironment cells are grown in also appears to be a critical factor as neural progenitor cells grown in proinflammatory conditions caused significant changes in RNA and protein levels with one study demonstrating enrichment of membrane bound IFN-γ and IFN-γ receptor 1 (IFNGR1) complexes that can induce key regulators of neuroinflammation and cell proliferation such as IFNGR1 and STAT1 (348). The employment of 3D printing could also be critical as 3D printing biomaterials will facilitate construction of complex precisely controlled microenvironments that could optimize EV production (319, 349, 350).

EVs are advantageous for therapeutic purposes as they protect nucleic acids and other bioactive components from degradation over long distances while also ensuring delivery to the cytosol of the target cell without triggering an auto-immune response (319). Although EVs hold great promise for transporting small interfering RNAs (siRNAs), miRNAs, mRNAs, and antisense oligonucleotides, in the case of some critical ncRNAs, the loading can be relatively low with as few as one bioactive copy of a given miRNA per EV (319, 351–353). Thus, it may be necessary to use high or repeated EV doses or employ cargo loading control strategies in situations where the dose of bioactive cargo is insufficient to adequately modulate gene expression (319, 351–353). Multiple studies have reported methods to load CNS-specific miRNA cargo into non-resident CNS cell types using genetic modification and subsequent over expression (14, 319). Other studies have used sonication, coincubation of EVs with desired cargo, and lipid or cholesterol functionalization (354–356).

One of the most important limitations of EVs to address for therapeutic purposes is the isolation protocols and scalability. Currently, several EV isolation strategies, each with their own advantages, have been utilized including differential centrifugation, ultrafiltration, precipitation, size exclusion chromatography, immunoisolation, and density gradient separation (357, 358). Ideally, an EV isolation protocol would be readily scalable to produce EVs with high purity for therapeutic purposes. The issue is that methods that result in a high purity can often have a relatively low yield and protocols with relatively high yields often co-isolate various protein aggregates, lipoproteins, and other impurities (357, 358). Therefore, the isolation protocol must always be optimized for yield and purity when manufacturing EVs for therapeutic purposes. Currently in the field, most groups are isolating EVs by differential centrifugation followed by ultracentrifugation (359). In situations where high purity is needed, further density-based purification using a sucrose or iodixanol centrifugation gradient can be used (360).

Finally, the route of administration appears to be a critical factor in EV therapy. More specifically, a 2021 study demonstrated that intralesional injection of EVs in a SCI rat model resulted in a more robust improvement of BBB score and sub-score compared to EVs delivered intravenously (288). Although intravenous injection is less invasive, delivering EVs intravenously will require higher doses of EVs as EVs can accumulate at off-target sites including the liver, spleen, and lungs, and systemic delivery of EVs intravenously results in delayed delivery of therapeutic EVs to the site of injury (288, 319, 333). The half-life of EVs is also an important to consider as a study observed a half-life of approximately 30 minutes *in vivo* in EVs loaded with dye after intravenous delivery (358). Therefore, intralesional injection could facilitate improved outcomes by more rapidly delivering a higher dose of therapeutic EVs to the lesion while minimizing off target accumulation and systemic degradation (7, 47, 48).

## 7 Discussion

SCI is a devasting event that has a complex pathophysiology. Following the primary mechanical injury, several secondary mechanisms of injury propagate cell dysfunction/cell death resulting in worse SCI patient outcomes. Among the many mechanisms of secondary injury, the systemic inflammatory response and neuroinflammation play pivotal roles in determining the severity of injury and patient outcomes. Following tSCI, the immune response likely exerts both beneficial and harmful roles and as previously discussed both the innate and adaptive immune systems have been implicated in secondary injury progression in tSCI. Thus, selectively targeting the immune response is a premier therapeutic approach as developing immunotherapeutics for SCI that shift the inflammatory cascade towards wound healing and away from secondary injury progression could drastically improve functional outcomes.

Recently, as discussed in this review, several studies have characterized the cytokine/chemokine cascades and the patterns of immune cell infiltration after SCI. Although there are some differences among humans, rats, and mice and further characterization is necessary, this work will facilitate therapeutic design and identification of potential biomarkers. Overall, TNFα, IL-1β, and IL-6 have been shown to be key mitigators of the immune response in the early hours/days following SCI. Although progress over the past two decades has significantly improved our understanding of the cytokine/chemokine cascades following tSCI, further characterization which identifies the timing of inflammatory cascades and the transit process of both innate and adaptive immune responses is necessary. More specifically, preclinical and clinical studies are needed that further characterize these inflammatory cascades and transit processes in the context of “beneficial” versus “harmful” responses to injury. A detailed molecular understanding of the inflammatory cascades underlying maladaptive injury responses versus wound healing responses would then allow for the design of novel immunotherapeutics or the repurposing of existing agents to shift maladaptive immune responses towards wound healing and thus potentially drastically improve outcomes. Over the next few decades, it is likely that immunomodulatory pharmaceuticals will be involved in managing SCI patients given the potential benefits. Due to the complex pathophysiology of SCI, however, multiple and synergistic immunotherapies may be required to promote recovery and halt maladaptive immune responses that promote secondary injury. In addition, clinical research and methods to improve targeted delivery to specific organs or cell types in a safe and stable manner will need to be developed and investigated.

Currently, there are multiple promising potential pharmaceutical agents and preclinical/clinical studies have reported no harmful effects and have suggested impressive benefits with NSAID/cyclooxygenase inhibitor, ChABC, and G-CSF therapy after SCI. Given these impressive improvements in functional results with no apparent harmful effects, we suggest a pressing need for clinical trials examining the role of these therapies in SCI. Two other pharmaceuticals, methylprednisolone and GM-1, appear to have minimal or questionable benefit in multiple studies and based off these studies should likely not routinely be used in SCI.

Furthermore, development of reliable SCI biomarkers that could predict outcomes and aid both medical management as well the surgical management of SCI patients is crucial. Due to the complicated pathophysiology underling SCI with likely both beneficial and detrimental immune responses following SCI, development of reliable predictive biomarkers has been challenging. Although recent studies have identified several proteins that appear to correlate with disease severity and patient outcomes including CSF IL-6, IL-8, tau, S100b, GFAP, and MCP-1, further studies are necessary in order to validate these proteins as true biomarkers that will guide clinical decision making.

Finally, given the complexity of SCI pathophysiology it is likely that these patients could benefit from not only immunotherapeutic agents but also combining other synergistic therapies including stem cell therapies/extracellular vesicle therapies and neuromodulation, spinal stimulation, and prosthetic devices which are beyond the scope of this review. In order to best apply combinations of these potentially synergistic therapies, further clinical and preclinical research investigating the efficacy, adverse effects, and biomarkers or parameters for reliable patient selection will be critical moving forward. In the case of stem cell transplantation, clinical trials have shown some promising results; however, overall, the results appear heterogenous. Further standardization including standardization between agencies will be necessary in the future. In addition, recent accumulating evidence suggests that the biological and therapeutic effects of stem cell therapy maybe due to secreted factors including EVs. Recent studies also suggest that EV therapy results in superior outcomes and is advantageous in that EV therapy mitigates concerns of immunogenicity and uncontrolled proliferation/differentiation that are associated with cellular transplants. Although the EV therapy has the potential to be revolutionary, several challenges as described in this review need to be addressed before it becomes a mainstream therapy. While challenges in developing pharmaceutical, stem cell, and EV therapies for SCI remain, new strategies and potential solutions continue to evolve which may provide a path forward to better the outcomes of SCI patients.

## Author contributions

RCS and RMS designed, wrote, edited, and approved the final version of the manuscript. RMS supervised the study. All authors contributed to the article and approved the submitted version.
